# Fatal balamuthosis in a Siberian tiger and a literature review of detection options for free-living amoebic infections in animals

**DOI:** 10.1177/10406387231160771

**Published:** 2023-03-12

**Authors:** Kevin D. Niedringhaus, Marissa Gordon, Michael J. Yabsley, Jackie Gai, Francisco A. Uzal, Kevin D. Woolard

**Affiliations:** Veterinary Medical Teaching Hospital, School of Veterinary Medicine, University of California–Davis, Davis, CA, USA; Wildlife Futures Program, Department of Pathobiology, School of Veterinary Medicine, University of Pennsylvania, Kennett Square, PA, USA; Veterinary Medical Teaching Hospital, School of Veterinary Medicine, University of California–Davis, Davis, CA, USA; Warnell School of Forestry and Natural Resources and Southeastern Cooperative Wildlife Disease Study, College of Veterinary Medicine, University of Georgia, Athens, GA, USA; Performing Animal Welfare Society, San Andreas, CA, USA; California Animal Health and Food Safety Laboratory, San Bernardino Branch, School of Veterinary Medicine, University of California–Davis, Davis, CA, USA; Department of Pathology, Microbiology, and Immunology, School of Veterinary Medicine, University of California–Davis, Davis, CA, USA; Department of Pathology, Microbiology, and Immunology, School of Veterinary Medicine, University of California–Davis, Davis, CA, USA

**Keywords:** amoeba, *Balamuthia* spp., meningitis, pneumonia, tigers

## Abstract

Free-living amoebae are rare causes of morbidity and mortality in humans and animals around the globe. Because the route of exposure and clinical progression of disease caused by different species of amoebae may vary in people and animals, determining the species of amoeba present is important. We describe here a fatal infection by the free-living amoeba *Balamuthia mandrillaris* in a Siberian tiger (*Panthera tigris altaica*). The 17-y-old patient had a rapid clinical decline after a peracute onset of severe lethargy, dull mentation, and anorexia. Autopsy did not identify a cause of death. Histology revealed inflammation associated with amoebic trophozoites in the brain, lungs, and iris of one eye. These amoebae were confirmed to be *B. mandrillaris* based on a PCR assay and sequencing. Although there are subtle morphologic differences between cyst stages of *Acanthamoeba* spp., *B. mandrillaris*, and *Naegleria fowleri* when present and identified on routine staining, other modalities, including PCR, immunofluorescence, electron microscopy, and immunohistochemistry, are typically utilized to confirm the pathogen involved in these cases. We review the reports of balamuthosis in animals.

Free-living amoebae have been implicated as causes of severe morbidity and mortality in animals on several continents.^16^ In humans, distinct nomenclature based on tissue distribution, pattern of inflammation, and the specific etiology have resulted in the designation of “primary amoebic meningoencephalitis” caused by *Naegleria fowleri* infection, “granulomatous amebic meningoencephalitis” caused by *Acanthamoeba* spp. or *Balamuthia mandrillaris* infections, and clinically less-severe “acanthamoebic keratitis” caused by one of several *Acanthamoeba* spp.^18^

Although this human terminology is often applied to animals, distinct clinical profiles based on the specific amoeba involved are less clearly defined in animals. As a result, determining the specific amoeba species present in these cases is important to further characterize and identify the potentially distinctive disease progression, risk factors, route of infection, organ distribution, and disease severity. Although there are morphologic differences among amoebae on routine histologic staining, the differences are subtle, require advanced magnification and visualization techniques, and involve examination of multiple life stages that are often less visible.

We describe here a case of fatal balamuthosis in a Siberian tiger (*Panthera tigris altaica*), provide a review of the literature on *B. mandrillaris* infections in animals, and describe the various detection options available to identify the causative amoeba. We did not include *Entamoeba* spp., which are important pathogens in reptiles, some amphibians, and primates, including people, given their differences in transmission, pathogenesis, and organs affected.^17^

A 17-y-old, castrated male, captive Siberian tiger that resided at a California exotic animal sanctuary since the age of 4 mo was presented because of acute onset of severe lethargy, dull mentation, and inappetence. The tiger was housed in a large habitat with grass, trees, a pool, and a den area, alongside 2 female siblings that appeared clinically normal. The animal was current on viral vaccines, including rabies (Imrab 3 TF; Merial), rhinotracheitis, panleukopenia, and calicivirus (Fel-O-Vax PCT + CaliciVax; Boehringer Ingelheim), and distemper (Live canarypox vector distemper vaccine; Merial). Bloodwork performed within 12 h before death revealed mild elevation of serum creatinine, mild hypokalemia, and a positive West Nile virus titer of ≥1:10 by SVN (Idexx), but was otherwise unremarkable. Significant medical history included severe keratitis and corneal rupture of both eyes suspected to be caused by felid herpesvirus 1 (FHV1; *Felid alphaherpesvirus 1*) infection requiring enucleation of the right eye and placement of a corneal graft on the left at 14-y-old. This tiger developed lordosis at a young age and more recently was diagnosed with severe degenerative spinal cord lesions. At 16-y-old, the tiger began showing progressively decreasing mobility and pelvic limb weakness. The tiger was being maintained on tramadol (1.5 mg/kg PO q12h), gabapentin (3 mg/kg PO q12h), and prednisolone (0.17 mg/kg PO q24h, later increased to 0.17 mg/kg PO q12h), as well as gastroprotectants and nutritional supplements to support joint function. Rapid clinical decline occurred, and, despite supportive care, the animal died ~18 h after presentation.

The tiger was submitted to the School of Veterinary Medicine at the University of California–Davis for postmortem examination. Gross examination confirmed intervertebral disc degeneration with secondary discospondylosis in the cervical, thoracic, and lumbar spinal column. Additional lesions included dozens of 1-mm, granular white nodules on the pleura of the right cranial pulmonary lobe as well as poorly defined, soft, ~1–3-cm masses in all remaining lobes of both lungs. Representative samples from all major organs, including eyes, trachea, lung, thyroid glands, tongue, heart, stomach, pancreas, small intestines, large intestines, liver, kidneys, spleen, adrenal glands, lymph nodes, spinal cord, and brain (cerebral cortex of frontal and parietal lobe, caudate nucleus, thalamus, hypothalamus, hippocampus, reticular formation, cerebellum, and brainstem) were collected, fixed in 10% neutral-buffered formalin, and processed routinely for histologic evaluation.

The most severe histologic lesion was found in the lungs and consisted of widespread, multifocal, suppurative inflammation within alveoli and alveolar septa, associated with abundant fibrin and fewer lymphocytes and histiocytes (Fig. 1). Surrounding many small vessels in these regions were variable numbers of round, 16–23-µm, foamy organisms consistent with amoeboid trophozoites (Fig. 2). The trophozoites contained 1 to rarely (<5%) 2 or 3 distinct, eosinophilic, 1–2-µm nucleoli. Abundant amoeboid trophozoites were also present in the meninges of the brain, being most abundant adjacent to the thalamus, and were associated with scattered lymphoplasmacytic inflammation. In addition to trophozoites, the meninges also contained many 8–11-µm, more dense and basophilic structures with a thin, slightly undulating outer membrane consistent with amoebic cysts (Fig. 3). Trophozoites with lymphoplasmacytic and histiocytic inflammation were also abundant within the iris of the left eye. Additional lesions unrelated to amoebic infection included moderate biventricular myocardial fibrosis, mild pancreatitis and gastritis, and minimal myelin sheath swelling with rare digestion chambers in the white matter of the brainstem. No significant abnormalities were observed in any of the other organs examined microscopically.

**Figures 1–3.**
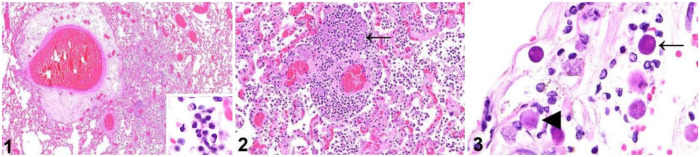
Tissues from a Siberian tiger infected with *Balamuthia mandrillaris*. **Figure 1.** Marked perivascular edema and alveolar infiltration by numerous inflammatory cells in the lung. Inset: higher magnification of the mostly neutrophilic infiltrate. H&E. **Figure 2.** Numerous round amoebic trophozoites (arrow) surround small vessels and are associated with neutrophils and fibrin in the lung. H&E. **Figure 3.** Amoebic trophozoites often have 1 or 2 prominent nucleoli (arrowhead) and are often accompanied by small basophilic amoebic cysts (arrow) in the meninges. H&E.

Selected sections of lung and brain were processed by immunohistochemistry (IHC) for *N. fowleri* (polyclonal; CDC R171), *Acanthamoeba* sp. (polyclonal; CDC R101), and *B. mandrillaris* (polyclonal; CDC R146) using pepsin for antigen retrieval as described previously.^6,11^ IHC for *B. mandrillaris* was strongly positive in the cytoplasm of the amoebae (Fig. 4A); there was less-intense cytoplasmic positivity for *Acanthamoeba* sp. (Fig. 4B). IHC for *N. fowleri* was negative (Fig. 4C).

**Figure 4.**
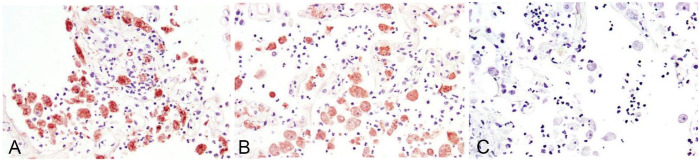
Immunohistochemical staining of amoebic organisms in the lung of a Siberian tiger with antibodies to (A) *Balamuthia mandrillaris*, (B) *Acanthamoeba* spp., and (C) *Naegleria fowleri*. Note that there is immunoreactivity to both *B. mandrillaris* (A) and *Acanthamoeba* spp. (B), although intensity is stronger for *B. mandrillaris*. There is no immunoreactivity to *N. fowleri* (C).

To clarify the slight immunohistochemical cross-reactivity between *Acanthamoeba* spp. and *B. mandrillaris* IHC, a PCR assay using primers AmeF977 and AmeR1534 targeting the 18S rRNA gene was used to differentiate these organisms.^13^ The consensus sequence was 97.2–97.9% similar to several *B. mandrillaris* sequences in GenBank. Based on a phylogenetic analysis conducted in MEGA X (https://www.megasoftware.net/), the tiger *B. mandrillaris* sequence grouped with several other *B. mandrillaris* from water, human, and mandrill (*Mandrillus sphinx*) samples from the United States and Mexico (Fig. 5).^18^ The sequence was deposited in GenBank (OQ236405).

**Figure 5.**
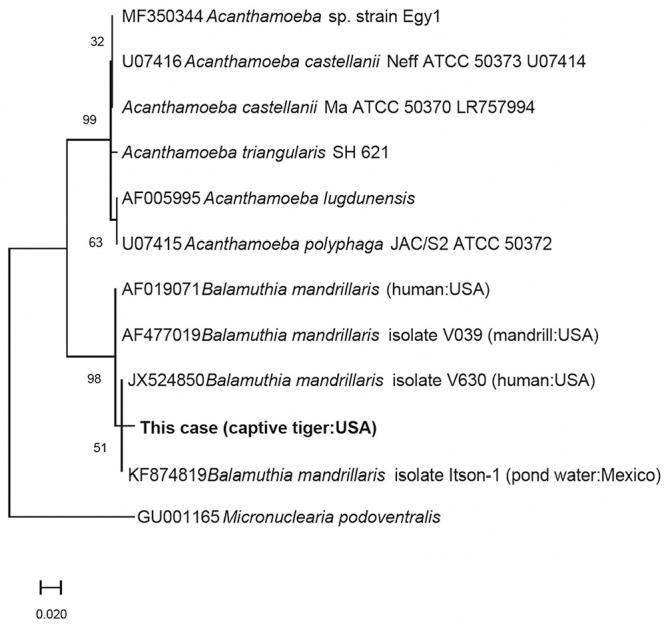
Phylogenetic tree constructed using the maximum-likelihood method and Tamura–Nei model comparing the *Balamuthia mandrillaris* organism sequenced in our report (in bold) to other sequences of selected free-living amoebae.

Although a wide variety of animals have been reported to develop severe disease associated with *B. mandrillaris* infection,^16^ we did not find another reported case in a felid after searching “balamuthia” and “tiger” in Google and PubMed. Fatal balamuthosis has been reported in humans worldwide^16^ and in at least 5 species of non-human primates in the United States,^8,9,15^ Germany,^14^ and Australia,^1^ in dogs in the United States^2,6^ and Australia,^5,10^ and in sheep,^7^ horses,^12^ and a flying fox in the United States.^3^ Most reports of balamuthosis are individual case reports, and the infections are associated with rare, sporadic mortality, particularly in immunosuppressed hosts. The exception is non-human primates, in which infection by *B. mandrillaris* is commonly implicated in mortality, particularly in gorillas.^8^ The reason for this is unclear. We did not find infection by B. mandrillaris in reptiles, amphibians, fish, or birds in searches of Google and PubMed.

In our case in a tiger, the potential role of exogenous steroids as a predisposing factor for the infection cannot be ruled out completely; however, this is unlikely given that the steroid dose at the time of death was low (0.17 mg/kg q24h, q12h) and not considered to be immunosuppressive. The historical keratitis and enucleated eye did not have histologic evidence of amoebic infection and was suspected to be caused by FHV1 infection. The route of infection in this case is unknown, but ingestion, contamination from a small wound, or inhalation are possible. Despite the potential for amoebae to infect eyes through the cornea, the uveitis in our case was likely the result of hematogenous spread.

The 4 main genera of pathogenic free-living amoebae can appear similar in histologic sections (Figs. 6–8) because many of the unique morphologic features are often only obvious in pure cell culture or via electron microscopy.^19^ However, some subtle differences can be detected histologically. Specifically, *B. mandrillaris* trophozoites are more pleomorphic than the other genera and can contain multiple nucleoli, which is considered species-specific (Fig. 7).^10,15^ The morphologic features of trophozoites and cysts, including overall size and number of nucleoli, in this tiger are consistent with those in other cases of balamuthosis in animals.^4,5,15^*Acanthamoeba* spp. trophozoites appear similar to the other groups and may have a cyst stage in tissue; the cysts have characteristic finger-like acanthopodia and have a more prominent, 2-layered cyst wall (Fig. 6 inset) compared to less-discrete 3 layers in *B. mandrillaris* (Fig. 7 inset), although these structures are more likely to be seen by electron microscopy or from isolation in culture.^16,20^*N. fowleri* has characteristic flagella, which are difficult to see in histologic sections, and lacks formation of a cyst stage in the brain (Fig. 8).^16,20^ Despite these morphologic differences, additional tests to confirm the pathogen are often warranted because these characteristic morphologic features can be subtle, particularly in biopsies or cases in which the intensity of infection is low, making cysts or multinucleate trophozoites challenging to identify. In animals, these tests primarily include immunoassays (IHC, immunofluorescence) and molecular techniques; however, isolation in culture and electron microscopy have also been used.

**Figures 6–8.**
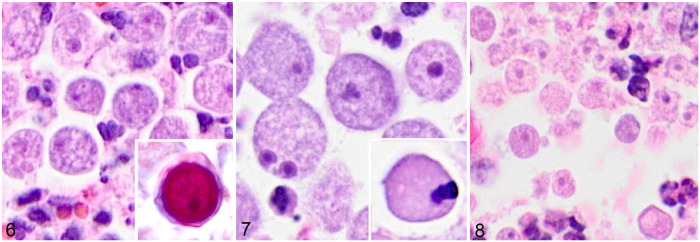
Histologic features of trophozoites and cysts of pathogenic free-living amoebae. **Figure 6.** Trophozoites of *Acanthamoeba* sp. in the lung of a horse. These trophozoites can look remarkably similar to those of *Naegleria fowleri*. Inset: *Acanthamoeba* sp. cyst from the brain of a human. Notice the readily visible, multi-layered, undulating outer membranes (Photo credit: Sarah Sapp, Parasitic Diseases Branch, Centers for Disease Control and Prevention; slides from different institutions result in variation in tinctorial staining). **Figure 7.** Trophozoites of *Balamuthia mandrillaris* in the lung of a horse. Note that one trophozoite has 2 nucleoli, a feature characteristic of this organism. Inset: cyst of *B. mandrillaris* in the lung of a horse. Notice the minimally undulating outer membrane and internal nucleoli. **Figure 8.** Trophozoites of *N. fowleri* in the brain of a cow. This organism characteristically lacks cysts in tissue sections.

Molecular methods have been used to identify *B. mandrillaris* in CSF in rare cases in the absence of direct observation,^8^ but PCR is also often negative even in severe cases of encephalitis in people.^20^ Direct visualization should be considered conclusive for amoebic infection in CSF and, although PCR can improve the sensitivity of detection when organisms are not observed, lack of detection from CSF should not rule out infection. Direct examination of CSF was not reported to be useful in any case of *B. mandrillaris* infection in animals; the inflammatory response detected in CSF is considered to be nonspecific for amoebic infections.^8,9^ Diagnosis of encephalitis from *B. mandrillaris* has not been made in biopsy samples from animals, to our knowledge, likely as a result of the clinical risk of obtaining samples in cases of encephalitis of presumed infectious origin.

IHC is commonly used to differentiate the free-living amoebae in tissues and utilizes described protocols.^3,4,11,12^ Curiously, there was cross-reactivity with *B. mandrillaris* and *Acanthamoeba* antibodies in our case (Fig. 4B), although the intensity of immunoreactivity was stronger for *B. mandrillaris* (Fig. 4A). IHC cross-reactivity was not reported in other cases of balamuthosis in the literature^3,6,11^ or in archived cases in our databases, but this technique may be useful in discriminating among these pathogens. The reason for cross-reactivity in our case is unclear. One report used IHC to highlight amoebic organisms but used antibodies that were not specific for a particular amoeba group.^8^ Histochemical staining was not used routinely to detect amoebic infections in animals; a few reports used Giemsa and/or periodic acid–Schiff to highlight the organisms in areas of severe inflammation,^8^ whereas others suggested that these stains did not adequately highlight the pathogens.^11^

Indirect immunofluorescence from tissue has been used to discriminate among *B. mandrillaris*, *N. fowleri*, and *Acanthamoeba* spp. in various species, and was the predominant modality found in our literature review of published cases.^1,3,5,7,10,11,14,15^ The protocols for using immunofluorescence are well-established,^11,19^ and cross-reactivity between amoebae was not reported in any of these studies.

Three reports used historical serum samples to detect antibodies to *B. mandrillaris* from apes via immunofluorescence of a reference stock of *B. mandrillaris* organisms or flow cytometry, but, to our knowledge, these are the only reported uses of serology to detect antibodies to free-living amoebae in animals.^4,9,14^ The study using a serologic assay in orangutans suggested that seroconversion was only reported in one animal with clinical disease, and others without disease were seronegative; however, formal validation of the assay for use in non-human species was not performed.^4^

A variety of molecular methods have been used to identify the causative agent in animals and most frequently targeted the 16S and 18S ribosomal RNA genes as well as the 40S ribosomal protein S11 and RNase P genes.^3,6,8,12,14^ To date, *B. mandrillaris* is the only known pathogenic species in the genus *Balamuthia* and, regardless of gene target used, *B. mandrillaris* can be distinguished from related amoebae. In animals, suspicion of amoebiasis may not occur until histology is performed. For these cases, molecular approaches can still be successful given that DNA extracted from formalin-fixed, paraffin-embedded tissue scrolls has been used successfully to amplify *B. mandrillaris*.^2,6^

Electron microscopy can be used to highlight the characteristic features of amoebae, but this modality was not reported widely in cases of *B. mandrillaris* infections in animals.^12^*Balamuthia* organisms can be present in either a 12–60-µm trophozoite with 1–2 nuclei with internal nucleoli or as a characteristic, triple-layered, pore-lacking cyst stage (Fig. 7).^15,19,20^ We speculate that the relatively small size of trophozoites in our case (16–23 µm) compared to some reports of *B. mandrillaris* trophozoites (50–60 µm) is that organisms expand or maintain their larger dimensions in culture, are smaller after formalin fixation, and, perhaps, shrink at lower tissue oxygen levels. Histologically, smaller (10–25 µm) trophozoites have been reported in other cases.^2,11^

Isolation of *B. mandrillaris* organisms in artificial media is less common compared to *N. fowleri* and *Acanthamoeba* spp., because *B. mandrillaris* is considered a soil amoeba and does not grow well on agar but will grow in living tissue culture cells including monkey kidney (E6) and human brain microendothelia and human lung fibroblasts.^20^ Additionally, *B. mandrillaris* has a lengthier generational time than *Acanthamoeba* or *N. fowleri*.^19,20^ Isolation was attempted in 2 reports of balamuthosis and was successful in a case from a horse^12^ but was unsuccessful in another because of overgrowth with bacteria despite the use of antibiotics.^15^ In both studies, isolation was attempted using established protocols,^19^ and, in the study from the horse, isolation was successful even after storage of infected tissue at −70°C for 60 d and was identified using indirect immunofluorescence. If isolation is successful, *B. mandrillaris* trophozoites have distinctive movement in culture that can be diagnostically useful; the motion is characterized by using finger-like pseudopodia or radiating unbranched pseudopodia resembling a spider.^19,20^
